# An Exome-seq Based Tool for Mapping and Selection of Candidate Genes in Maize Deletion Mutants

**DOI:** 10.1016/j.gpb.2018.02.003

**Published:** 2019-02-08

**Authors:** Shangang Jia, Kyla Morton, Chi Zhang, David Holding

**Affiliations:** 1 Department of Agronomy and Horticulture, Center for Plant Science Innovation, Beadle Center for Biotechnology, University of Nebraska, Lincoln, NE 68588, USA; 2 School of Biological Sciences, Center for Plant Science Innovation, Beadle Center for Biotechnology, University of Nebraska, Lincoln, NE 68588, USA

**Keywords:** Maize functional genomics, Mapping-by-sequencing, Exome-seq, Opaque mutant, Bulked segregation analysis

## Abstract

Despite the large number of genomic and transcriptomic resources in maize, there is still much to learn about the function of genes in developmental and biochemical processes. Some maize mutants that were generated by gamma-irradiation showed clear segregation for the kernel phenotypes in B73 × Mo17 F2 ears. To better understand the functional genomics of kernel development, we developed a mapping and gene identification pipeline, bulked segregant exome sequencing (BSEx-seq), to map mutants with kernel phenotypes including opaque endosperm and reduced kernel size. BSEx-seq generates and compares the sequence of the exon fraction from mutant and normal plant F2 DNA pools. The comparison can derive mapping peaks, identify deletions within the mapping peak, and suggest candidate genes within the deleted regions. We then used the public kernel-specific expression data to narrow down the list of candidate genes/mutations and identified deletions ranging from several kb to more than 1 Mb. A full deletion allele of the *Opaque-2* gene was identified in mutant 531, which occurs within a ∼200-kb deletion. **Opaque mutant** 1486 has a 6248-bp deletion in the mapping interval containing two candidate genes encoding RNA-directed DNA methylation 4 (RdDM4) and AMP-binding protein, respectively. This study demonstrates the efficiency and cost-effectiveness of BSEx-seq for causal mutation mapping and candidate gene selection, providing a new option in **mapping-by-sequencing** for **maize functional genomics** studies.

## Introduction

Maize (*Zea mays*) has been used extensively as a model organism for genetics and developmental studies. Seed mutants of maize are especially attractive for crop researchers because the ordered rows of kernels, held in place on the ears, allows easy identification of genetically-segregating kernel phenotypes. For instance, the well-characterized *opaque-2* (*o2*) mutant possesses increased levels of essential amino acids lysine and tryptophan [1], and the identified gene encodes the endosperm-specific basic leucine zipper domain (bZIP) transcription factor O2. Being part of a complex regulatory network containing prolamine-box binding factor (PBF) and O2 heterodimerizing proteins (OHP1 and OHP2), O2 is involved in the biosynthesis of zein proteins and starch [2], [3]. In addition, many other genes causing opaque phenotypes in maize mutants [4] have been identified, such as *opaque1* [5], *floury1* [6], *floury2* [7], *defective endosperm B30* [8], and *floury4* [9]. It is likely that characterization of new opaque mutants would lead to the identification of more genes involved in endosperm maturation.

**Supplementary Figure S1:**
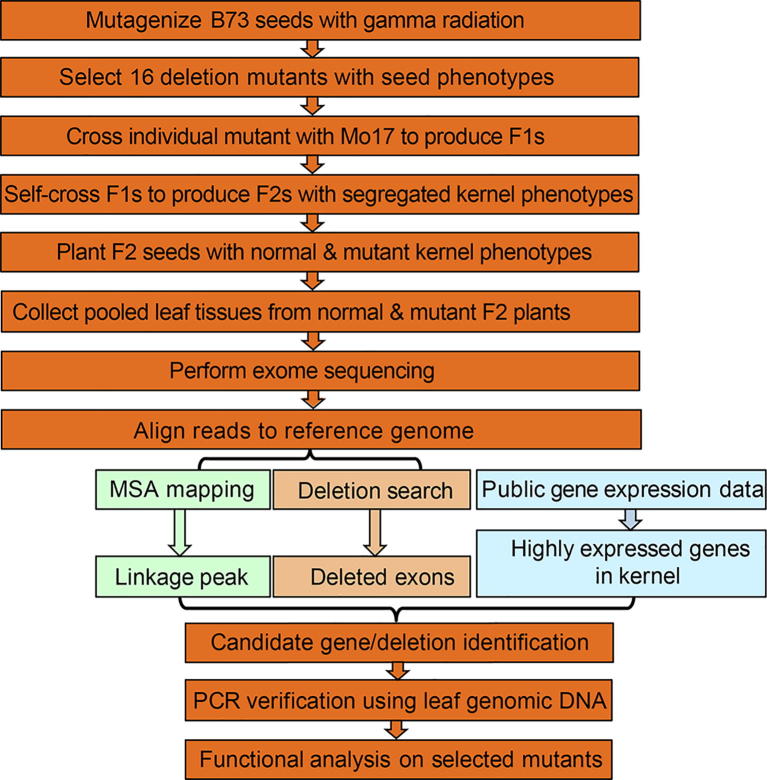
**BSEx-seq work flow**

Radiation mutagenesis is known to create maize mutants in forward genetics studies [10]. Compared to point mutations caused by ethyl methane sulfonate (EMS) and insertional and deletional mutagenesis caused by transposons, radiation often causes deletions [11], [12], [13], which permanently removes partial or whole genes, as well as inducing inversions, duplications, and translocations [14], [15]. Our previous studies using γ-radiation and exome sequencing (exome-seq) have demonstrated the key role of 27-kD γ-zein in restoration of a vitreous kernel phenotype in Quality Protein Maize [12]. Moreover, by combining bulked segregant RNA sequencing (BSR-seq) and exome-seq, we previously developed BSREx-seq to map and identify causal genes/deletions involved in seed development in the B73 background [13]. In the present study, we further streamlined the mapping strategies and improved the cost-effectiveness. We report the development and performance evaluation of bulked segregant exome sequencing (BSEx-seq) in a new batch of B73-deletion mutants with kernel phenotypes. Instead of separating the M2 exome-seq from F2 BSR-seq, BSEx-seq uses only exome-seq of normal and mutant F2 DNA pools to obtain the linkage peaks based on single nucleotide polymorphisms (SNPs), and calls deleted exons within the mapping intervals (Figure S1). Linkage peaks in BSEx-seq were identified using mapping-by-sequencing analysis (MSA), a software package developed in our lab [16]. MSA is available in the GitHub repository (https://github.com/jsg200830/Mapping-by-Sequencing-Analysis).

## Results

### BSEx-seq allows simultaneous mapping and selection of candidate genes in maize kernel deletion mutants

**Figure 1:**
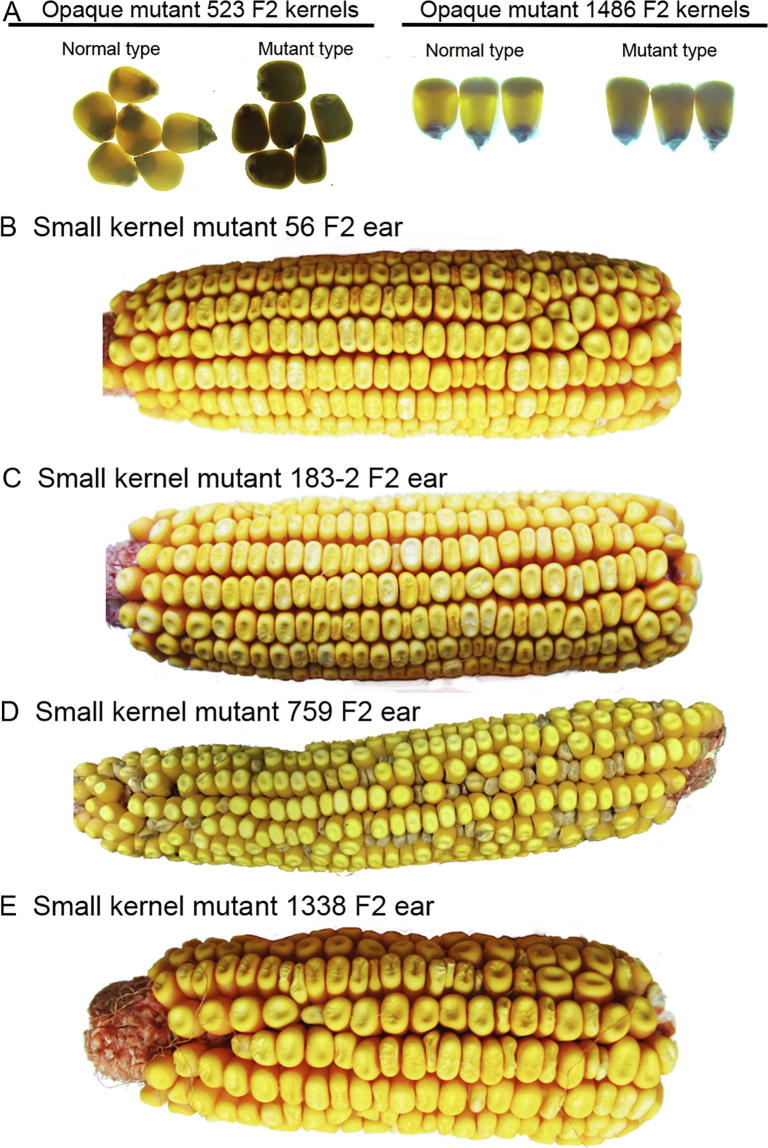
**Kernel phenotypes from segregating F2 ears** **A.** F2 kernels showing segregation for opaque mutants 523 and 1486. F2 ears showing segregation for small kernel mutants 56 (**B**), 183-2 (**C**), 759 (**D**), and 1338 (**E**), respectively.

**Supplementary Figure S2:**
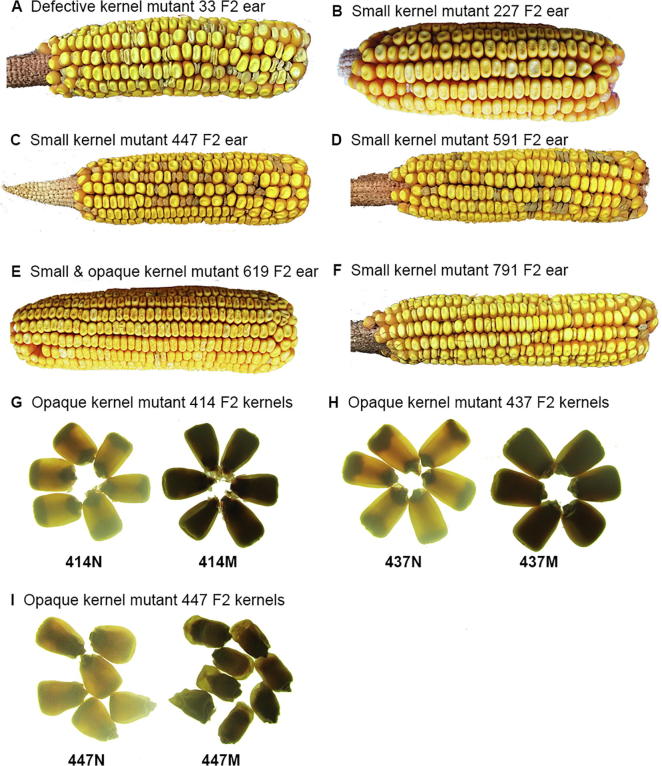
**Kernel phenotypes in mutants 33 (A), 227 (B), 447 (C), 591 (D), 619 (E), 791 (F), 414 (G), 437 (H), and 447 (I)**

BSEx-seq conducts a linkage analysis by comparing SNPs between normal and mutant DNA pools. In this study, we focused on opaque endosperm and reduced kernel size phenotypes which, along with other mutant classes, were numbered according to a common scheme (Table 1). Mutants 414, 437, 523, 531, and 1486 have normal-sized opaque kernels, that light cannot penetrate on a light box (Figure 1 and Figure S2). The reduced kernel size phenotype varies widely, ranging from subtle small kernel in mutants 56, 183-2, and 227, to severe defective small kernel in mutants 759, 33, 591, 791, and 1168. Mutants 1338, 447, and 619 exhibit both small kernel and opaque endosperm phenotypes. Since some mutants are found to be sterile as homozygotes due to pollen or ear defects, we collected young leaves from the 2-week-old F2 plants of all mutants for DNA extraction. All seeds used to grow the F2 plants for a particular mutant were from one single segregating ear (Figure 1 and Figure S2).

**Table 1:** **Summary of maize mutants with kernel phenotypes identified using BSEx-seq in this study**

**Mutant ID**	**Kernel phenotype**	**Location of linkage peak**	**Deletion (kb)**	**Best candidate genes**
33	Defective kernel shrunk	5: 191 Mb	TBD	TBD

56	Subtle small kernel	4: 135 Mb	∼1500	GRMZM2G160174/Zm00001d051033 (*autophagy-related protein*)

183-2	Subtle small kernel	6: 156 Mb	∼538	TBD

227	Subtle small kernel	6: 131 Mb	TBD	TBD

414	Large opaque	6: 152 Mb	TBD	TBD

437	Large opaque	1: 276 Mb	TBD	TBD

447	Small kernel opaque	2: 1.26 Mb	TBD	TBD

523-1	Large opaque	1: 46 Mb	∼1200	GRMZM2G443655-GRMZM2G073628/Zm00001d028726 (*protein disulfide isomerase-9*)

531	Large opaque	NA	∼200	GRMZM2G015534/Zm00001d018971 (*opaque-2*)

591	Small kernel	7: 21 Mb	TBD	TBD

619	Small kernel opaque	2: 29.5 Mb	TBD	TBD

759	Severe small kernel	10: 87 Mb	∼480	GRMZM2G060987/Zm00001d024815 (*Nop53 domain protein*)

791	Severe small kernel	5: 20 Mb	TBD	TBD

1168	Small kernel	7: 168.6 Mb	TBD	TBD

1338	Small kernel opaque	1: 145 Mb	∼3500	GRMZM2G118743/Zm00001d030598 (*aberrant lateral root formation 4*), GRMZM2G045668/Zm00001d030573 (*transcription initiation factor IIE*)

1486	Large opaque	10:1.86 Mb	∼6.248	GRMZM2G098603/Zm00001d023237 (*RNA-directed DNA methylation 4*), GRMZM2G098596-GRMZM2G176546/Zm00001d023238 (*AMP binding protein*)

*Note*: Location of linkage peak refers to the location for summit of linkage peak and is indicated with chromosome number and genomic coordinates; for example, 1: 46 Mb in mutant 523 indicates the summit of this linkage peak is located at genomic coordinate 46 Mb on chromosome 1. The best candidate genes refer to the genes with the highest expression values in endosperm and embryo (FPKM > 50). Gene IDs in both RefGen_v3.25 and RefGen_v4 are listed with the corresponding gene names provided in the parenthesis. Some other genes with high expression in endosperm and embryo may also be considered as candidate genes but are not included in this table. TBD, to be determined; NA, not available.

More than 98% of the 190,641 exons in the maize genome were covered with largely comparable read mapping depth in BSEx-seq. The average sequencing depth (*i.e.*, *Dn*; see Methods) in 16 mutants using BSEx-seq is around 20, ranging from 15.38 to 29.26 (Table S1), indicating that it produced sufficient sequencing depth. We called around 400,000 SNPs/indels using BSEx-seq reads in a comparison of normal and mutant pools in the 14 mutants, except for mutants 531 and 1168. To lower the noise caused by sequencing error and natural variation between inbreds B73 and Mo17, only the SNPs/indels from one of F2 parents of B73 and Mo17 were kept, based on the publically available genome sequence data (B73, SRA accession No: SRR910231 for B73 and SRA accession No: SRR764595 for Mo17). And we determined >1000 positive SNPs/indels in all the mutants except for mutant 531, which are inherited from B73 in the mutant pool against the SNPs/indels from Mo17. The use of the reference set of SNPs/indels based on the parent genome sequences (B73 and Mo17) helped decrease the false positive SNPs in mapping analysis, since they were cross checked among normal pool, mutant pool, Mo17, and B73. The number of positive SNPs/indels can be high (up to 11,938 in mutant 759; Table S1), although an extremely low number was detected in mutant 531 (only 3 positive SNPs/indels). We then plotted the positive SNPs/indels in a window size of 100 kb to identify linkage peaks across the whole genome, and obtained linkage peaks from 15 out of 16 mutants examined (Table 1). We further defined the range of linkage peaks, which are distributed in one single specific chromosome, and inferred the candidate causal genes based on the location of the summit of the peaks (Table 1). We failed to generate a linkage peak for mutant 531, due to the insufficient number of SNPs detected, and this was the result of a cross of M1 generation to B73 wild type not Mo17. However, even without a mapping population in mutant 531, the exome-seq still enabled us to identify a new full deletion allele of *opaque-2* created by the mutagenesis.

We identified deletion candidates in the mutant pools, based on the ratio of *Dnn*/(*Dnm* + 0.05) with a cutoff of 30, which may be responsible for the phenotype if they were located underneath the linkage peaks detected. The size of deletions associated with the mapping peak varied from 6248 bp to 3.5 Mb, and these sizes were verified using genomic PCR based on the absence of an amplicon using the DNA extracted from a mutant plant (Table 1). We then downloaded gene expression data from qTeller database (http://www.qteller.com), which were annotated based on BLAST searches for sequence similarity, and selected the gene candidates based on their relatively higher expression in endosperm and/or embryo (FPKM > 50). Consequently, we were able to select 1–2 candidate genes in six mutants (Table 1).

BSEx-seq allows construction of linkage peaks across the whole genome, making it easier to identify the causal gene, in comparison with BSR-seq (Table 2). A relatively lower average coverage (<20) was obtained and only 70% of exons were found to be expressed and could thus be used for SNP calling in BSR-seq (Table S1). These data indicate that >30% exons are not expressed or detectible in leaf RNA using BSR-seq. Moreover, read coverage varied among mutants when using BSR-seq (with Dn ranging from 9.42 to 19.09). Therefore, BSEx-seq is able to produce more SNPs/indels and identify more positive SNPs (thousands), while BSR-seq only generated hundreds of positive SNPs (Table S1). Furthermore, BSR-seq is always used to make linkage peaks, and it is difficult to call a deletion with BSR-seq data alone, because genes are often not expressed in the mutant and an absence of reads in exons does not directly indicate a deletion candidate.

**Table 2:** **Feature comparison between BSEx-seq and BSR-seq**

	**BSEx-seq**	**BSR-seq**
In common	Two pools	Two pools
	SNP calling	SNP calling
	Linkage peak	Linkage peak
	Only coding region covered	Only coding region covered

Advantage	Based on DNA sequencing	Based on RNA sequencing
	Covering all genes	Only covering expressed-genes
	More SNPs	Fewer SNPs
	Identification of deletions	Identification of null-expression genes only
	Leaf or any tissue	Tissue of interest preferred
	Comparable coverage for genes at different expression levels	Fewer SNPs for low-expression genes

Disadvantage	No expression data	Expression data in mutants

### Candidate gene identification without a mapping population

**Figure 2:**
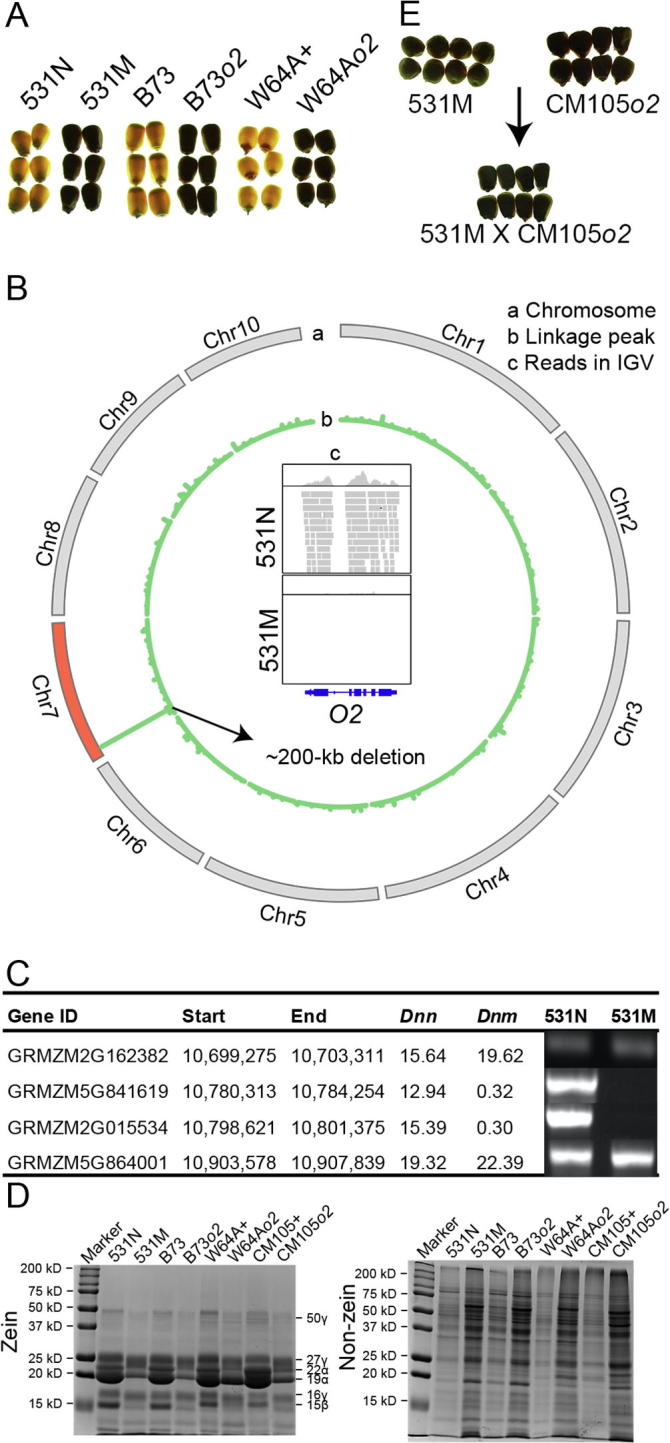
**A novel *o2* deletion allele in opaque mutant 531** **A.** Vitreous kernels that light can penetrate *vs.* opaque kernels that light cannot penetrate for mutant 531 in F2s, B73 *vs.* B73*o2*, and W64A + *vs.* W64A*o2*. **B.** Chromosomal plotting of deletion ratios in mutant 531. Ten chromosomes were shown in the outer circle, with chromosome 7 in red for causal deletion found (a). Deletion ratios were calculated according to the definition *Dnn*/(*Dnm* + 0.05) by comparing normal and mutant F2 pools in BSEx-seq and shown in the inner circle (ranging 0 to 152,240, b). *O2* gene on chromosome 7 showed no reads in the mutant pool, compared to the normal pool in IGV software (c). **C.** Genomic PCR analysis confirming that two genes GRMZM5G841619 and GRMZM2G015534 were deleted, whereas the other two neighboring genes (GRMZM2G162382 and GRMZM5G864001) were not deleted in 531M. *Dnn* and *Dnm* indicate the normalized BSEx-seq read depth for normal and mutant samples, respectively. **D.** Coomassie staining showing the zein and non-zein protein profiles for 531N, 531M, B73, B73*o2*, W64A+, W64A*o2*, CM105+, and CM105*o2*. **E.** Allelism test for *o2* deletion allele with 531M × CM105*o2*, all with opaque kernels. o2, opaque 2; N, normal; M, mutant.

Mutant 531 exhibits a classical opaque kernel phenotype, similar to other *opaque-2* lines, such as W64A*o2*, B73*o2*, W22*o2*, and CM105*o2* (Figure 2A). This mutant was crossed to B73 wild type instead of Mo17. As a result, we only identified a very low amount of both SNPs/indels and the positive ones (Table S1), resulting in a failure in generating linkage peaks in mutant 531. However, based on the read coverage ratio *Dnm* between normal and mutant pools, we identified a deletion (∼200 kb) on chromosome 7, which contains three genes (GRMZM5G841619 encoding the tryptophan synthase alpha subunit 1, AC208337.2_FG003 annotated as chromatid cohesion protein SCC2 and GRMZM2G015534 encoding *O2*, shown as the peaks in the layer b of Figure 2B. The *O2* gene was deleted in its entirety in the mutant pool, with no reads mapped in the mutant but many reads recovered in the normal control as shown in the layer c of Figure 2B. Genomic PCR analyses further confirmed this deletion with no amplified PCR products in the two border genes, GRMZM5G841619 and GRMZM2G015534 (Figure 2C).

**Supplementary Figure S3:**
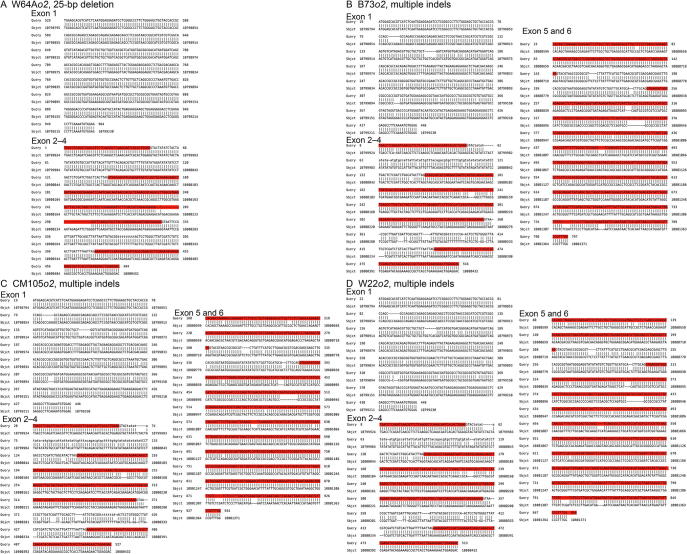
**The *o2* alleles in W64A*o2* (A), B73*o2* (B), CM105*o2* (C), and W22*o2* (D) mutants** Exons are highlighted in red. “Query” refers to the sequences of *o2* alleles, and “Sbjct” refers to the sequence corresponding to *o2* alleles in the maize reference genome (RefGen_v3.25), based on the BLAST results.

To compare with the other typical *o2* mutants, we conducted an SDS–PAGE analysis for zein and non-zein protein profiles. It showed that mutant 531 contained an obviously reduced amount of zein proteins (especially α-zeins and 15kD β-zeins), and increased amount of non-zein proteins; protein characteristics of other *o2* lines (Figure 2D). In order to confirm that the deletion in mutant 531 was allelic to other *o2* mutations, we performed allelism tests by crossing mutant 531 with CM105*o2.* The mutants failed to complement each other and the F1 seeds exhibited an opaque phenotype, confirming *O2* as the causal gene (Figure 2E). Sequencing of other *o2* mutant alleles revealed two classes of mutations. The first class has a 25-bp deletion and includes W64A*o2*, and the second class has multiple indels and includes B73*o2*, W22*o2*, and CM105*o2* (Figure S3). Thus, mutant 531, with a full deletion of the *O2* gene, is a new null allele*.*

### Mapping and candidate gene identification in opaque mutants

**Figure 3:**
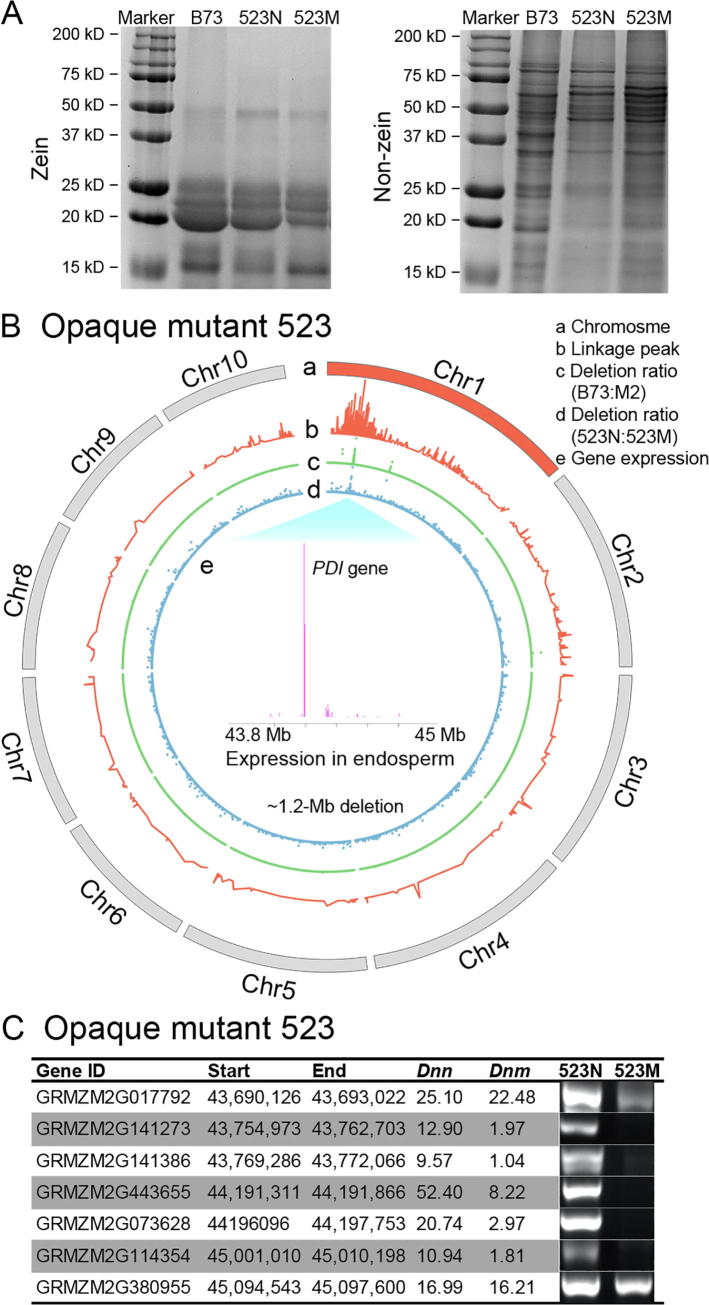
**Candidate gene and causal deletion search in opaque mutant 523** **A.** Coomassie staining showing the zein and non-zein protein profiles in opaque mutant 523 (523 N and 523 M refer to the normal and opaque mutant kernels in F2s, respectively). **B.** Chromosomal plotting in mutant 523. a, ten chromosomes, with chromosome 1 in red for causal deletion found; b, linkage peak (ranging from 0 to 24); c, deletion ratio (ranging from 0 to 5792) based on *Dn_(B73)_*/(*Dnm* + 0.05) in a comparison of B73 and M2 plants using exome-seq; d, deletion ratio (ranging from 0 to 390) based on *Dnn*/(*Dnm* + 0.05) in a comparison of normal and mutant F2 pools using BSEx-seq; e, gene expression values (FPKM) of 25DAP in endosperm in the ∼1.2-Mb deletion [34], the *PDI* gene (Zm00001d028726) with the highest expression level was selected as candidate gene. **C**. Genomic PCR analysis showing that five genes were deleted in the deletion region, and two neighboring genes (GRMZM2G017792 and GRMZM2G380955) were not deleted. *Dnn* and *Dnm* indicate the normalized BSEx-seq read depth for normal and mutant samples, respectively.

In opaque mutant 523, the content of zein proteins was obviously decreased in comparison to wild type B73 kernels, while the content of non-zein proteins was increased, same as in the *o2* mutants (Figure 3A). The content of all zein proteins in mutant 523 was reduced more generally. This is different from *o2* mutants, which show more specific reduction in the content of 19-kD and 22-kD α-zein proteins as well as the 15-kD β-zein. To dissect this opaque mutant for causal mutation and gene candidate responsible for the aforementioned phenotype and protein profiling, we used BSEx-seq analysis. We found that positive SNPs/indels intensively appeared on chromosome 1, and the highest linkage peak was identified with a summit at the genomic coordinate 46.05 Mb as shown in the circle b of Figure 3B, indicating a linkage disequilibrium with causative mutation. Furthermore, we identified a large deletion (∼1.2 Mb) with significant read coverage difference between the mutant and normal pools as shown in the circle d of Figure 3B, which is exactly underneath the summit of linkage peak and considered as causative deletion candidate.

To search for deletions using inbred plants as with the exome-seq component of BSREx-seq instead of hybrid (B73 × Mo17) F2s, we also performed exome-seq in M2 mutant plants and compared with a B73 wild type control. Our results showed that the ∼1.2-Mb deletion was identified at the same location as shown in the circle c of Figure 3B. This indicates that BSEx-seq using hybrid DNA has a similar capacity to inbred DNA in identifying the causal deletion candidates. The causal deletion candidate in mutant 523 covers 130 exons in 26 genes. We selected five genes, including two boundary genes and three random genes interspersed throughout the deletion. Using primers designed inside the exons, genomic PCR analysis with leaf DNA as template confirmed that all five genes tested were deleted, as shown by the absence of PCR products in mutant 523, compared to the normal plant control. In contrast, the two neighboring genes beyond the two ends of this deletion were shown not to be deleted, which clearly indicated the range of this deletion (Figure 3C). Although multiple genes are included in this causal deletion, expression of most of these genes in endosperm is very low. In contrast, two genes (GRMZM2G443655 and GRMZM2G073628), which are collapsed into one single gene Zm00001d028726 in B73 genome assembly RefGen_v4, showed high expression in endosperm (Figure 3B, layer e). Zm00001d028726 is the only gene in this large deletion with endosperm-specific expression (https://www.maizegdb.org/gene_center/gene/GRMZM2G073628, and https://www.maizegdb.org/gene_center/gene/GRMZM2G443655), suggesting it as a strong candidate gene for the opaque phenotype (Table 1). Interestingly, in the NCBI BLAST nt database, this gene is annotated as protein disulfide isomerase 9 (PDI9), which is one of several classes of chaperones intricately involved in storage protein accumulation in the endosperm [17], and the amino acids of GRMZM2G443655 and GRMZM2G073628 matched its two domains.

**Figure 4:**
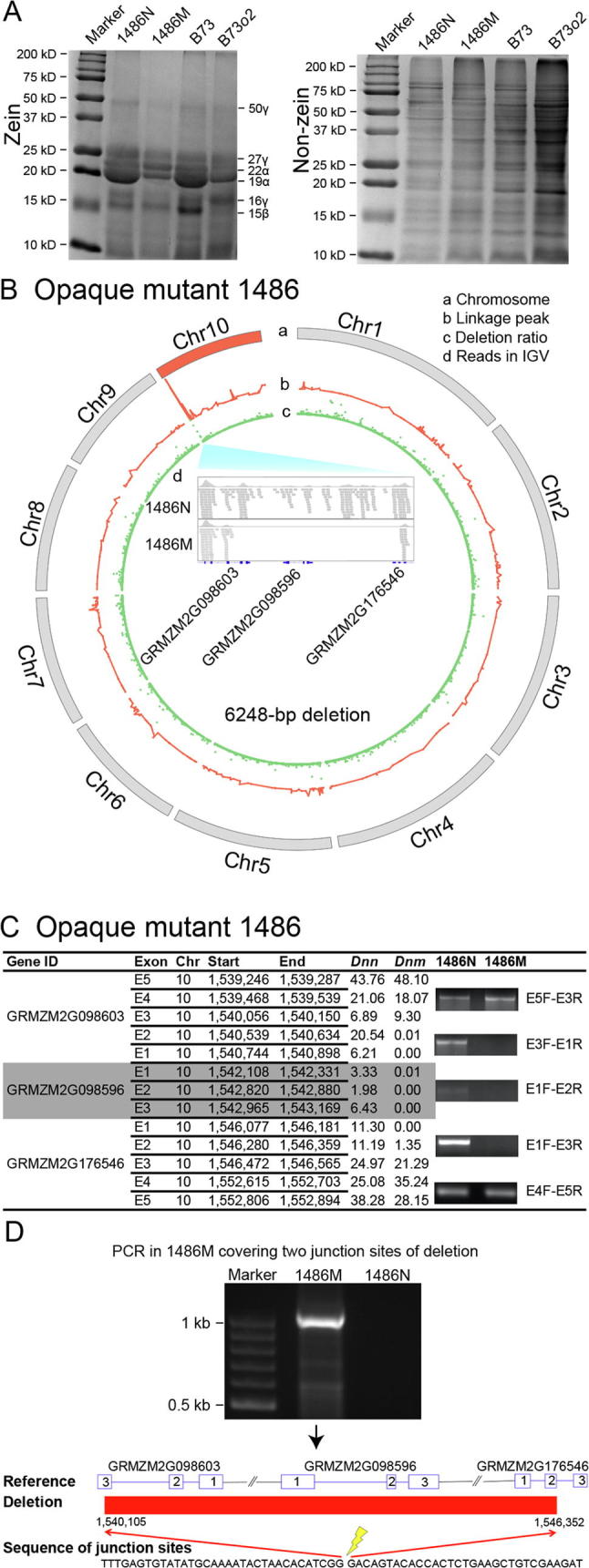
**Causal gene candidate search in opaque mutant 1486** **A.** Coomassie staining showing the zein and non-zein protein profiles in comparison with those of B73 and B73*o2*. **B.** Chromosomal plotting in mutant 1486. a, ten chromosomes, with chromosome 10 in red for causal deletion found; b, linkage peak (ranging from 0 to 30) in red; c, deletion ratio (ranging from 0 to 268) based on *Dnn*/(*Dnm* + 0.05) in a comparison of normal and mutant F2 pools in BSEx-seq; d, in the deletion region, no reads were found in the mutant pool, compared to the normal pool in IGV software. **C.** Genomic PCR showing that three genes were located within the deletion. Exons are shown for the three genes, with “E” followed by exon number. Primer pairs are listed beside the respective PCR products. *Dnn* and *Dnm* indicate the normalized BSEx-seq read depth for normal and mutant samples, respectively. **D.** Genomic PCR analysis using primer pair of GRMZM2G098603_E5F and GRMZM2G176546_E3R showing presence of a ∼1-kb band using DNA from mutant sample but not from normal sample. Clone sequencing analysis of this PCR product confirmed the 6248-bp deletion and the two break points at 1,540,105 and 1,546,352 based on the sequence information of maize B73 genome assembly v3.25.

Mutant 1486 is also a classical (normal sized kernel) opaque kernel mutant (Figure 1). However, zein profiling revealed a general reduction in zein protein content and little or no compensatory increase in the content of the non-zein proteins (Figure 4A), indicating a different mechanism in conferring the opaque phenotype. Using BSEx-seq, we identified a sharp linkage peak as shown in the circle b of Figure 4B and a candidate for causal deletion as shown in circle c of Figure 4B at the beginning (genomic coordinate 1.86 Mb) of chromosome 10. This included three genes as shown in IGV software (GRMZM2G098603, GRMZM2G098596, and GRMZM2G176546) in the center (Figure 4Bd). No reads were recovered from this deletion region in the mutant pool. These observations were confirmed by genomic PCR analyses. We designed five primer pairs to cover the three genes. The PCR analysis showed that amplification of three genomic PCR products failed in the mutant, compared to the normal control, whereas the other two PCR products were amplified successfully in both normal and mutant DNA samples and suggested the border of the deletion (Figure 4C). These data indicated that the middle gene GRMZM2G098596 and partial exons in the other two border genes (GRMZM2G098603 and GRMZM2G176546) were within this ∼6-kb deletion.

To further dissect the deletion, we designed primers (GRMZM2G098603-E5F and GRMZM2G176546-E3R) within the non-deleted exons in the two outer genes, and amplified a ∼1-kb PCR product, which spans the junction site when using genomics DNA obtained from mutant 1486 but is too large (>7 kb) to amplify when using DNA obtained from normal plants (Figure 4D). Sequencing of this product provided the precise genomic coordinates of two break points at 1,540,105 and 1,546,352, resulting in an actual deletion size of 6248 bp (Figure 4D). Genome-wide structural variations called by Lumpy [18] also identified this deletion and two break points supported by 2 paired-end and 12 single-end reads. These data confirmed that, in total, eight exons in the three genes were deleted. Among them, two genes (GRMZM2G098596 and GRMZM2G176546) are annotated to encode AMP binding protein, and collapsed into one single gene Zm00001d023238 in B73 genome assembly RefGen_v4. The other gene (GRMZM2G098603 or Zm00001d023237) is annotated to encode an RNA-directed DNA methylation 4 gene (RdDM4), based on the BLAST search.

### Large deletions can be mapped but candidate gene selection is more challenging

**Figure 5:**
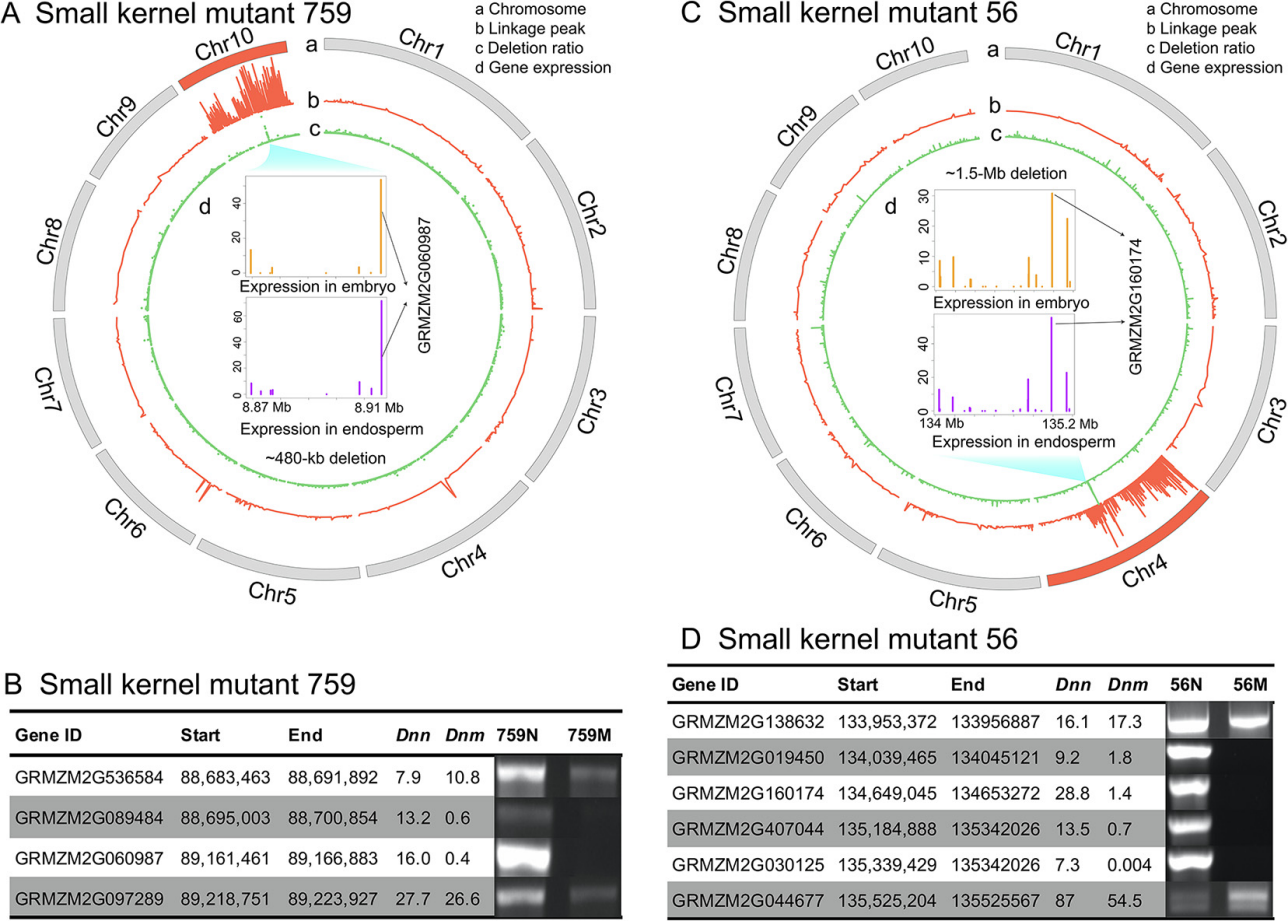
**Causal deletion search in small kernel mutants 759 and 56** **A.** Chromosomal plotting in mutant 759. a, ten chromosomes, with chromosome 10 in red for causal gene/deletion found; b, linkage peak (ranging from 0 to 73) in red; c, deletion ratio (ranging from 0 to 904) based on *Dnn*/(*Dnm* + 0.05) in a comparison of normal and mutant F2 pools in BSEx-seq; d, gene expression values (FPKM) of 25DAP in endosperm and embryo [34] in the deletion are shown, and the gene (GRMZM2G060987) with significantly higher expression is likely the candidate gene. **B.** Genomic PCR showing that two neighbor genes (GRMZM2G089484 and GRMZM2G060987) were deleted around the border of the deletion region in mutant 759, whereas the other two neighboring genes are not deleted. *Dnn* and *Dnm* indicate normalized BSEx-seq read depth for normal and mutant samples, respectively. **C.** Chromosomal plotting in mutant 56. a, ten chromosomes, with Chromosome 4 in red for causal gene/deletion found; b, linkage peak (ranging from 0 to 47) in red; c, deletion ratio (ranging from 0 to 778) based on *Dnn*/(*Dnm* + 0.05) in a comparison of normal and mutant F2 pools using BSEx-seq; d, gene expression values (FPKM) of 25DAP in endosperm and embryo [34] in the deletion are shown, and the gene (GRMZM2G160174) with the highest expression is the likely candidate gene. **D.** Genomic PCR analysis showing that four genes were deleted in the deletion region in mutant 56, whereas two neighbor genes (GRMZM2G138632 and GRMZM2G044677) were not deleted. *Dnn* and *Dnm* indicate normalized BSEx-seq read depth for normal and mutant samples, respectively. DAP, day after pollination.

Small kernel mutants (Figure 1) represent a broad and variable phenotypic group with many potential genetic causes. Diffuselydistributed linkage peaks were found on chromosome 10 in the severe small kernel mutant 759 (circle b, Figure 5A) and on chromosome 4 in the subtle small kernel mutant 56 (circle b, Figure 5C), causing the failure in inferring a specific region with causal deletion and candidate genes. However, by comparing the sequencing coverage using BSEx-seq, we were able to identify a large deletion within the mapped chromosome.

In small kernel mutant 759, a single ∼480-kb deletion was detected spanning 88,686,064–89,166,883 on chromosome 10. There are 45 exons in this deletion, making up nine genes likely including the causal gene (circle c, Figure 5A). This deletion was confirmed using genomic PCR analysis by amplification of the four neighboring genes through the two ends of the deletion, among which two neighboring genes in the deletion were shown to be deleted, whereas the other two outside of the deletion were not (Figure 5B). GRMZM2G060987, which encodes a prenylated Rab acceptor 1 (PRA1) family protein, could be a candidate gene responsible for the phenotype in small kernel mutant 759, considering its high expression in embryo and endosperm in wild type.

In the subtle small kernel mutant 56, a single ∼1.5-Mb candidate causal deletion was identified on chromosome 4 (circle c, Figure 5C), which contained 77 exons making up 19 genes. PCR of selected dispersed genes within this deletion confirmed the deletion borders (Figure 5D). Public expression profiles from qTeller database (http://www.qteller.com) showed that the gene GRMZM2G160174 is highly expressed in both embryo and endosperm (d in the center, Figure 5C), whereas other genes in the deletion had low seed-specific expression. These data suggest that GRMZM2G160174, which encodes an autophagy-related protein, could be a candidate gene responsible for the phenotype in small kernel mutant 56.

**Supplementary Figure S4:**
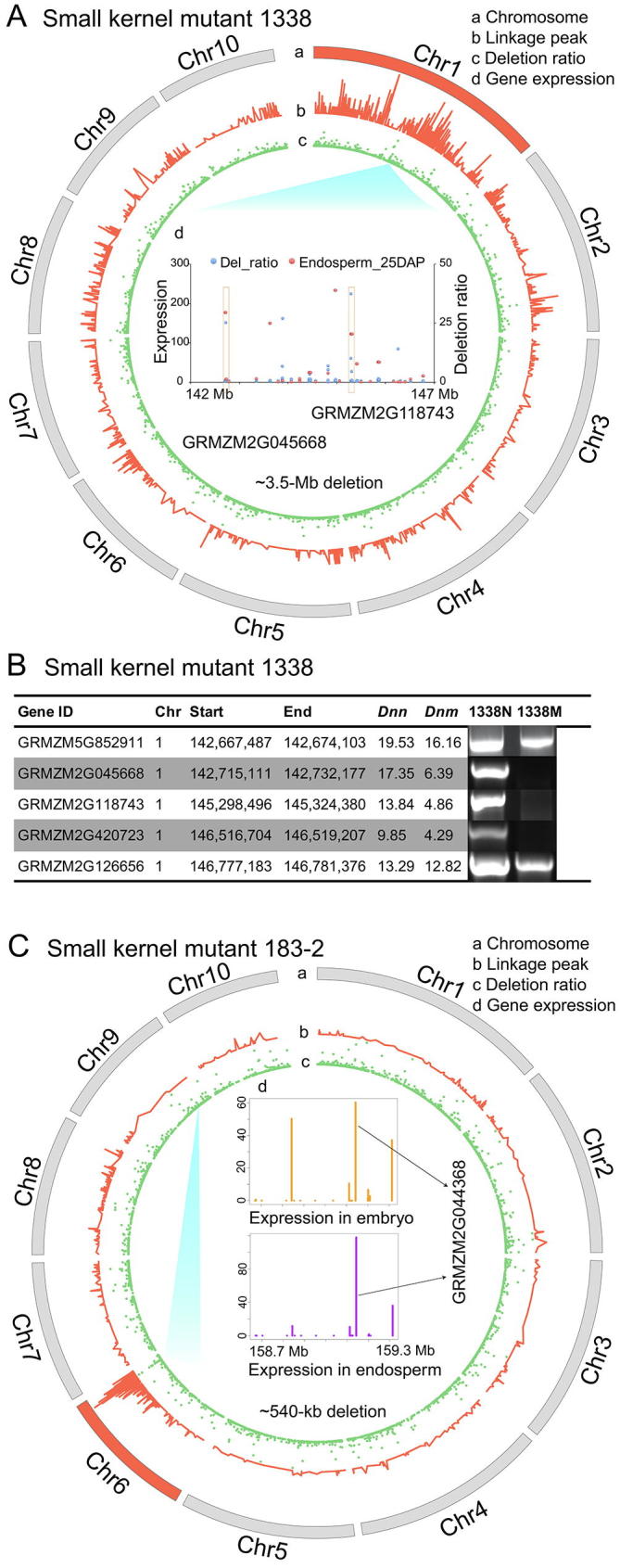
**Causal gene search in mutants 1338 and 183-2 with small kernels A.** Chromosomal plotting in mutant 1338. a, ten chromosomes in mutant 1338, with chromosome 1 in red for causal gene/deletion found; b, linkage peak (ranging from 0 to 16) in red; c, deletion ratio (ranging from 0 to 268) based on *Dnn*/(*Dnm* + 0.05) in a comparison of normal and mutant F2 pools in BSEx-seq; d, gene expression values (FPKM) of 25DAP in endosperm and deletion ratio of *Dnn*/(*Dnm* + 0.05) in the deletion are shown, and the genes (GRMZM2G118743 and GRMZM2G045668) with higher values are the likely candidate genes. **B**. Genomic PCR analysis shows that three genes (GRMZM2G045668, GRMZM2G118743, and GRMZM2G420723) were deleted in the deletion region in mutant 1338, whereas the two neighbor genes (GRMZM5G852911 and GRMZM2G126656) were not deleted. *Dnn* and *Dnm* indicate the normalized BSEx-seq read depth for normal and mutant samples, respectively. **C**. Chromosomal plotting in mutant 183-2. a, ten chromosomes in mutant 183-2, with chromosome 6 in red for causal gene/deletion found; b, linkage peak (ranging from 0 to 25) in red; c, deletion ratio (ranging from 0 to 210) based on *Dnn*/(*Dnm* + 0.05) in a comparison of normal and mutant F2 samples using BSEx-seq; d, gene expression values (FPKM) of 25DAP in endosperm and embryo in the deletion are shown, and the gene (GRMZM2G044368) with higher expression is the likely candidate gene.

Multiple genes are lost in large deletions, many of which are functionally unknown. For instance, in small kernel mutant 183-2, we identified a linkage peak at the genomic coordinate 155,560,000 on chromosome 6 (Figure S4C). A region with enriched deleted exons was located around 159 Mb. In this region, there are multiple possible candidate genes that showed high expression in kernel. Therefore, more than one gene may contribute to the phenotype when the deletions span multiple genes. For example, in mutant 1338, two genes, GRMZM2G045668 and GRMZM2G118743, are deleted within the linkage peak on chromosome 1, both of which have high expression in endosperm (Figure S4A and S4B). In such cases, analysis of additional mutant alleles for genes independently and in allelism crosses is the only way to resolve causality.

## Discussion

### Advantages and disadvantages of BSEx-seq

We previously developed BSREx-seq by combining BSR-seq for linkage peaks and exome-seq for deletion identification [13]. However, despite its efficiency in identifying positive variations and making linkage peaks, BSREx-seq requires two rounds of sequencing; RNA-seq in F2s and exome-seq in M2s, thus incurring considerable cost. The BSR-seq component of BSREx-seq allows mapping of genes with kernel-specific expression (not expressed in leaves) even when leaf RNA is used because SNPs can be derived from neighboring genes that are expressed in the leaves. However, BSR-seq using leaf RNA is usually not useful for inferring potential loss of expression of a candidate gene since the causal gene is often not expressed in leaves. To overcome the aforementioned limitation of BSREx-seq, here we developed BSEx-seq and evaluated its performance for mapping maize deletion mutants and identifying candidate genes using a single sequencing dataset. Firstly, just like BSR-seq, BSEx-seq requires two pools of plants (normal and mutant), and makes use of coding genes’ sequences and high-density SNPs for linkage peaks. Since BSEx-seq uses leaf DNA samples, deleted exons can be identified in the mapping interval whether they are expressed or not. More importantly, BSEx-seq utilizes only one sequencing dataset for obtaining linkage peaks and identifying causal deletion candidate genes, while BSR-seq can only be used to make linkage peaks (Table 2). Furthermore, the Zeanome array included almost all the exons in the maize genome assembly v3.25 (more than 98% of 190,641 exons), and also some other unknown genes. BSR-seq relies on expressed genes, and the genomic regions with linkage disequilibrium may have many genes without detectable expression. Therefore, the number of SNPs is much higher in BSEx-seq than BSR-seq (Table S1). In this study, we are able to generate linkage peaks from almost all the mutants. A sharp linkage peak was obtained, for example, in mutant 1486 and the putative causal deletion is only ∼300 kb away from the summit of linkage peak. Compared to the whole genome sequencing, the most significant advantage of BSEx-seq is cost-effectiveness, due to the enriched sequencing. In summary, BSEx-seq is efficient for mapping and searching for candidate genes in deletion mutants.

**Supplementary Figure S5:**
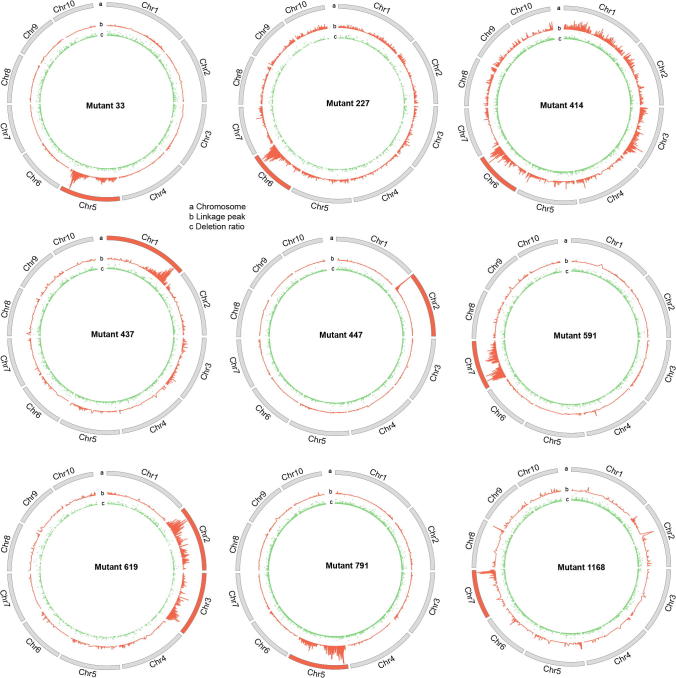
**Linkage peaks and deletion identification in nine opaque and small kernel mutants** Linkage peaks and deletion identification are shown in mutants 33 (red layer, linkage peak ranging from 0 to 65; green layer, deletion ratio based on *Dnn/*(*Dnm*+0.05) in a comparison of normal and mutant F2 samples ranging from 0 to 184), 227 (red layer, linkage peak ranging from 0 to 80; green layer, deletion ration ranging from 0 to 168), 414 (red layer, linkage peak ranging from 0 to 47; green layer, deletion ration ranging from 0 to 284), 437 (red layer, linkage peak ranging from 0 to 29; green layer, deletion ration ranging from 0 to 192), 447 (red layer, linkage peak ranging from 0 to 79; green layer, deletion ration ranging from 0 to 272), 591 (red layer, linkage peak ranging from 0 to 46; green layer, deletion ration ranging from 0 to 188), 619 (red layer, linkage peak ranging from 0 to 30; green layer, deletion ration ranging from 0 to 163), and 791 (red layer, linkage peak ranging from 0 to 64; green layer, deletion ration ranging from 0 to 386). The circle layers are the same to those in Figure S4.

Despite the advantages mentioned above, there are several limitations in BSEx-seq. First, similar to BSREx-seq, while it can broadly map deletions, BSEx-seq cannot define deletion coordinates if the deletions reside in the non-coding regions. In this study, we have identified one or more candidates for causal deletions in six mutants. However, some mutants do not suggest candidate mutations despite the often very sharp linkage peaks (Figure S5). Such mutations may occur in regulatory regions or may result from translocations, duplications, or inversions [14], [15]. In maize, gene regulatory regions may be substantially separated from their own coding regions; as a result, such deletions could not be identified using BSEx-seq or BSR-seq. A second disadvantage is that DNA sequencing from exon data does not provide any expression data as does BSR-seq. To generate expression data, conventional RNA-seq experiments for selected mutants using developing kernel tissue are needed. Such expression data are useful to show expression changes of genes in the genomic regions where linkage peaks are located, and provide more clues for identification of causal genes. In this study, the public expression data from qTeller database in endosperm and embryo was used for filtering out candidate genes, which reduced the cost substantially. However, RNA-seq on developing kernels would be needed to aid in candidate gene selection, when such data are not available. Another disadvantage is that BSEx-seq relies heavily on the exon capture methodology and reagents, both of which confer considerable cost compared with a BSR-seq alone strategy.

### Identification of structural variation and break points of deletion

Structural variations (SVs), such as insertions, deletions, and duplications, can be identified using next-generation sequencing. There are several ways to detect large SVs, including *de novo* assembly, read splitting, inconsistencies of insert sizes through paired-end reads, and read coverage depth analysis [19]. Multiple software tools are available for this use, for example, Pindel [19], SVseq2 [20], Delly [21], Lumpy [18], and Sprites [22]. These tools heavily reply on splitting breakpoint-containing reads and discordant read pairs, to determine the breakpoints of deletions. Here, BSEx-seq focused on the coding regions, while the deletions or other possible SVs caused by gamma-radiation occurred randomly in the whole genome. If breakpoints were outside of coding regions, it is impossible to discover any SVs due to lack of reads in the intergenic regions. For example, in mutant 523, no SVs were found by Lumpy within the mapping interval on coordinate 46 Mb of chromosome 1, while our read coverage depth analysis showed a deletion of ∼1.2 Mb, which was confirmed by genomic PCR analysis (Figure 3). Similar situations were observed in mutants 56 and 759, *i.e.*, deletions detected by our BSEx-seq (Figure 5) were not identified by Lumpy, indicating that their break points are not within exons. However, if the breakpoints were within exons, it is possible to make use of these SV searching tools. For example, the deletion in mutant 1486 broke exon 3 of gene GRMZM2G098603 and exon 2 of gene GRMZM2G176546, and Lumpy software easily identified a deletion of 6248 bp and the genomic coordinates of two break points, which were confirmed by our genomic PCR and sequencing analyses (Figure 4). Actually, this is the only case with SVs in the mapping interval detected by Lumpy in these 16 mutants. In BSEx-seq, the available SV search tools can be helpful and supplemental to our perl script of deletion identification based on read depth analysis.

### Identifying candidate genes

With falling sequencing costs and increasing multiplexing capabilities, we show the potential to perform mapping of many mutants simultaneously using exome-seq of mutant and wild type pools in a single Illumina HiSeq 2500 experiment. This is not possible using whole-genome mapping-by-sequencing strategies without genome enrichment. Using BSEx-seq, we generated linkage peaks in 15 mutants and identified candidate deletions/genes in five of them. We previously generated and characterized a new *opaque-1* allele in opaque mutant 937, resulting from a 6.2-kb intra-gene deletion [13]. Here, we reported a new *opaque-2* allele within a larger deletion in opaque mutant 531. Therefore, isolation of additional alleles of already cloned mutants may suggest that we are getting close to finding all the gene products needed to condition a vitreous endosperm phenotype. However, we have also described new classical opaque mutants such as 1486 and 523. Further biological characterization would likely yield new insights into the process of endosperm maturation.

Loss of many gene products could condition an opaque phenotype during the synthesis, transport, and modification of storage proteins and starch. It is therefore worthwhile to characterize new opaque mutants. For example, one candidate gene in mutant 523 encodes a protein disulfide isomerase (PDI) molecular chaperone. Chaperones play an important role in protein folding in the endoplasmic reticulum [23]. Maize PDI-like ZmPDIL5-1 accumulates during endoplasmic reticulum stress [24]. In maize, PDI accumulates at high levels in seeds to produce storage proteins under normal growth conditions [24], [25]. Some known maize opaque mutants showed that PDI was involved in triggering induction of the ER stress and unfolded protein response. For example, in *fl2*, *Mc*, and *DeB30*, PDI and ER binding protein (BiP) work together as molecular chaperones, and their expression increases in response to dominant negative mutations in zein genes [8], [26], [27]. Whether or not PDI is the causal gene in mutant 523, its reduced zein, increased non-zein phenotype may result in increased kernel lysine, like in the *opaque-2*. Mutant 1486 has opaque kernels and a general reduction in zein proteins, but unlike *o2*, it does not have substantially increased non-zein proteins. The 6248-bp deletion within the mapping interval contains two candidate genes, encoding RdDM4 and AMP binding protein, respectively. Investigation of phenotypes of available UniformMu transposon insertion alleles and complementation tests should be able to reveal the causal gene.

**Supplementary Figure S6:**
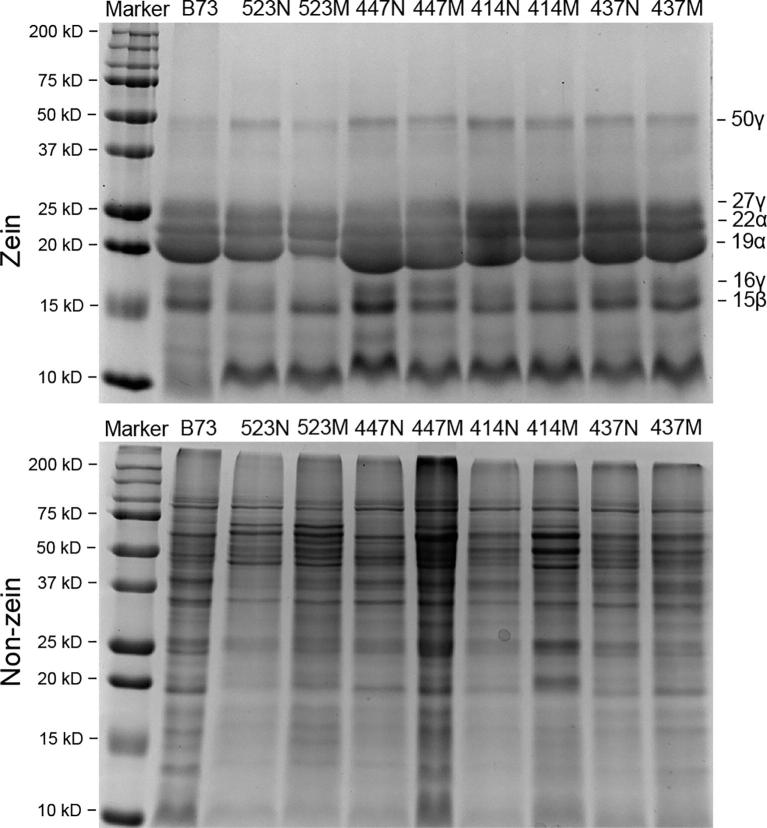
**Zein and non-zein protein profiles in opaque mutants** Normal and mutant kernels from F2 ears with segregation were used for zein and non-zein extraction.

Although good linkage peaks were obtained in mutants 33, 227, 414, 437, 447, 591, 619, 791, and 1168 (Figure S5), causal deletions or candidate genes cannot currently be assigned. These mutants may be caused by loss or alteration of regulatory elements in the non-coding genomic regions. Mutants 531, 523, and 1486 all have reduced content of zein proteins, through different modes of action to condition an opaque endosperm phenotype. However, in mutants 414 and 437, zein levels are normal, and in mutant 447, they are only slightly reduced (Figure S6). As has been illustrated with mutants like *opaque-1* [5], mutants that result in opacity through a zein-independent mechanism are useful for a global understanding of endosperm maturation. In general, these mutants represent a useful resource for maize researchers for a better understanding of endosperm development.

## Materials and methods

### Sample collection

The B73 maize mutants were created as previously described [13]. Sixteen mutants were selected based on the kernel phenotype (Table 1), and F1s were generated by outcrossing M2s with Mo17. F2 ears with segregating kernel phenotype were harvested from the field in 2015 (Figure 1 and Figure S2). For each mutant, 30 normal seeds and 30 mutant seeds were planted in small pots. Leaves from 14-day-old plants in the same group were cut and pooled together for DNA extraction. DNA was extracted and purified as described previously [13], before sending out for exome-seq.

### Exome-seq

The SeqCap EZ Developer Maize Exome kit (Roche NimbleGen, Madison, WI) was used to enrich ∼110-Mb exon regions, including 39,621 genes and 190,641 exons in B73 annotation (RefGen_v3.25). Briefly, four columns in one SeqCap EZ Developer Maize Exome kit were used to process 32 samples (16 pairs of normal and mutant sample pools from 16 mutants) with bar coding, based on the online protocol (http://sequencing.roche.com/products/nimblegen-seqcap-target-enrichment/seqcap-ez-system/seqcap-ez-developer.html). Exon-enriched DNA fragments were used to construct Illumina sequencing libraries, and, 32 samples were pooled into two lanes for sequencing on HiSeq 2500, with a 125-bp paired-end run and v4 chemistry. All exome capture and sequencing were performed in the University of Minnesota Genomics Center. The exome-seq data were deposited in the Short Reads Archive (SRA) of NCBI (accession No: SRP067758).

### Linkage analysis

The analysis method previously developed for BSREx-seq was optimized to perform the linkage analysis in BSEx-seq [13]. Raw reads were trimmed with Trimmomatic v0.36 [28] to make sure the average quality score larger than 28 and having the minimum length of 110 bp. The trimmed reads were then mapped against the B73 reference genome (RefGen_v3.25) using Bowtie2 v2.2.4 [29] with the default parameters. All mapping files were processed to generate pair-wise pileup files by Samtools (v1.4) [30]. Variants (SNPs and indels) were called using VarScan v2.3.7 [31] in the command “mpileup2cns” with the default parameters. Only the sites covered by more than five reads in each sample were used for SNP/indel calling. A reference set of polymorphisms was built by analyzing the downloaded whole genome sequencing datasets for B73 (SRA accession No: SRR910231) and Mo17 (SRA accession No: SRR764595). We further compared the SNPs/indels in every mutant–normal pair to this reference set to obtain positive SNPs/indels. Positive SNPs/indels are defined as the ones which are homozygous in the mutant pool and identical with that of B73 in reference SNPs/indels set, but heterozygous in the normal pool with alleles from Mo17 genome. The advantage of using this reference SNP/indel set between B73 and Mo17 is to potentially exclude the sequencing error, mutations by gamma-radiation, and variants inside inbred B73 and Mo17. Chromosomal recombination and linkage disequilibrium enrich positive SNPs/indels in the region with the causal gene. Positive SNPs/indels were plotted in a window of 100 kb in the CIRCOS software v0.67 [32]. The linkage peaks were then identified using MSA [16], which was designed to identify the association regions showing linkage disequilibrium to mutant phenotype and place linkage peaks on the chromosomes.

### Identification of causal deleted exon candidate genes using BSEx-seq

Gamma radiation creates mutations randomly. In this study, we focused on identification of deleted exons. A deletion in exonic regions could be discovered if significant differences of DNA read coverage were found between the normal and the mutant pools. The accumulated depth (*D*) for a given exon was the sum of sequencing depth of all positions in this exon. The normalized depth (*Dn*) of an exon was calculated as (10^9^ × *D*)/(*LD_a_*), where *D_a_* is the total accumulated depth of the whole genome and *L* is the exon length.

To identify deleted exons, two perl scripts were developed to calculate *Dn*, including *Dnn* for the normal pool and *Dnm* for the mutant pool, and the ratio of *Dnn*/(*Dnm* + 0.05) for deletion candidates, based on the bam depth files by Samtools. Both scripts are available with detailed running command lines at GitHub (https://github.com/jsg200830/Mapping-by-Sequencing-Analysis). To assist the determination of the break points of deletion, structural variations were called using Lumpy [18], based on the alignment files of pair-end reads by Speedseq [33].

We searched the candidate deletions in two steps, *i.e.*, screening the deleted genes underneath the linkage peak, and determining candidate genes with high expression in developing kernels using public tissue-expression data. First, we screened deletions of genes/exons based on read coverage, and genes underneath linkage peaks were considered as candidates. This strategy works well when only a single gene is involved in the causative deletion. However, a large deletion under the linkage peak region could include multiple genes, which can be identified by exome-seq. To further determine candidate genes in multigenic deletions, we downloaded the tissue expression data from qTeller database (http://www.qteller.com). qTeller collated RNA-seq datasets and provides normalized expression for different tissues, especially the endosperm and embryo at 25 days after pollination (DAP) [34] in this study. The genes with the highest or specific expression in the kernel or endosperm in the mapping interval are considered to be more likely causal gene candidates, especially when other nearby genes are not expressed in the kernel.

### Validation of causal deletions using genomics PCR analysis

After using BSEx-seq to map causal genomic regions and deleted exons therein, PCR analyses of deleted candidate genes were conducted with genomic DNA and primers designed based on the exon sequences in B73 (Table S2). Single seeds with normal or mutant kernel phenotypes from a single segregating F2 ear were planted in green house. After 2 weeks, leaf tissues were collected for extraction and purification of genomic DNA as described previously [13]. PCR cycling conditions for all primer pairs were as follows: initial denaturation at 95 °C for 2 min; 30 cycles of 94 °C for 30 s, 60 °C for 30 s, and 72 °C for 2 min; and 72 °C for 10 min, using Taq DNA polymerase (New England Biolabs, Ipswich, MA). Successful amplification of PCR products using DNA from both the normal and the mutant plants indicates no deletion, whereas the failed amplification from the mutant sample suggests potential deletion. To verify the border of a deletion identified by BSEx-seq, the neighboring genes outside of the deleted region and the bordering genes inside the deleted region were selected for PCR amplification. Sequencing of PCR products spanning the deletion can be used to confirm the two junction sites when the deletion is short (several kb).

### Zein protein profiling analysis

Total proteins including zein and non-zein fractions were extracted from mature endosperm of single kernels from F2 ears as described previously [35]. Briefly, total proteins were extracted from 50 mg kernel flour using a sodium borate (pH 10) extraction buffer containing 2% 2-mercaptoethanol and 2% SDS. Zein proteins were separated from non-zein proteins by selective precipitation of non-zein proteins in 70% (v/v) ethanol. Zein and non-zein fractions from equivalent amounts of flour were separated using SDS–PAGE. Gels were stained with Coomassie blue for protein visualization.

## Authors’ contributions

DH conceived the study, and participated in the study design and coordination. CZ participated in the design of the study and data analysis. SJ carried out the molecular genetic studies and conducted the data analysis. KM participated in the mutant population creation and field work. SJ and DH wrote the manuscript with the help from CZ. All authors read and approved the final manuscript.

## Competing interests

The authors have declared no competing interests.

## References

[1] Mertz E.T., Bates L.S., Nelson O.E. (1964). Mutant gene that changes protein composition and increases lysine content of maize endosperm. Science.

[2] Yang J., Ji C., Wu Y. (2016). Divergent transactivation of maize storage protein zein genes by the transcription factors Opaque2 and OHPs. Genetics.

[3] Zhang Z., Zheng X., Yang J., Messing J., Wu Y. (2016). Maize endosperm-specific transcription factors O2 and PBF network the regulation of protein and starch synthesis. Proc Natl Acad Sci U S A.

[4] Hunter B.G., Beatty M.K., Singletary G.W., Hamaker B.R., Dilkes B.P., Larkins B.A. (2002). Maize opaque endosperm mutations create extensive changes in patterns of gene expression. Plant Cell.

[5] Wang G., Wang F., Wang G., Wang F., Zhang X., Zhong M. (2012). *Opaque1* encodes a myosin XI motor protein that is required for endoplasmic reticulum motility and protein body formation in maize endosperm. Plant Cell.

[6] Holding D.R., Otegui M.S., Li B.L., Meeley R.B., Dam T., Hunter B.G. (2007). The maize *floury1* gene encodes a novel endoplasmic reticulum protein involved in zein protein body formation. Plant Cell.

[7] Coleman C.E., Clore A.M., Ranch J.P., Higgins R., Lopes M.A., Larkins B.A. (1997). Expression of a mutant α-zein creates the *floury2* phenotype in transgenic maize. Proc Natl Acad Sci U S A.

[8] Kim C.S., Hunter B.G., Kraft J., Boston R.S., Yans S., Jung R. (2004). A defective signal peptide in a 19-kD α-zein protein causes the unfolded protein response and an opaque endosperm phenotype in the maize *De*-B30* mutant. Plant Physiol.

[9] Wang G., Qi W., Wu Q., Yao D., Zhang J., Zhu J. (2014). Identification and characterization of maize *floury4* as a novel semidominant opaque mutant that disrupts protein body assembly. Plant Physiol.

[10] Nannas N.J., Dawe R.K. (2015). Genetic and genomic toolbox of *Zea mays*. Genetics.

[11] Sato Y., Shirasawa K., Takahashi Y., Nishimura M., Nishio T. (2006). Mutant selection from progeny of gamma-ray-irradiated rice by DNA heteroduplex cleavage using *Brassica petiole* extract. Breed Sci.

[12] Yuan L., Dou Y., Kianian S.F., Zhang C., Holding D.R. (2014). Deletion mutagenesis identifies a haploinsufficient role for gamma-zein in *opaque2* endosperm modification. Plant Physiol.

[13] Jia S., Li A., Morton K., Avoles-Kianian P., Kianian S.F., Zhang C. (2016). A population of deletion mutants and an integrated mapping and exome-seq pipeline for gene discovery in maize. G3 (Bethesda).

[14] Mizuno H., Kawahigashi H., Ogata J., Minami H., Kanamori H., Nakagawa H. (2013). Genomic inversion caused by gamma irradiation contributes to downregulation of a *WBC11* homolog in *bloomless* sorghum. Theor Appl Genet.

[15] Belfield E.J., Gan X., Mithani A., Brown C., Jiang C., Franklin K. (2012). Genome-wide analysis of mutations in mutant lineages selected following fast-neutron irradiation mutagenesis of *Arabidopsis thaliana*. Genome Res.

[16] Jia S., Zhang C., Holding D. (2017). A mapping-by-sequencing tool for searching causative genes in mutants. IEEE Int Conf Electro Inf Technol.

[17] Galili G., Sengupta-Gopalan C., Ceriotti A. (1998). The endoplasmic reticulum of plant cells and its role in protein maturation and biogenesis of oil bodies. Plant Mol Biol.

[18] Layer R.M., Chiang C., Quinlan A.R., Hall I.M. (2014). LUMPY: a probabilistic framework for structural variant discovery. Genome Biol.

[19] Ye K., Schulz M.H., Long Q., Apweiler R., Ning Z. (2009). Pindel: a pattern growth approach to detect break points of large deletions and medium sized insertions from paired-end short reads. Bioinformatics.

[20] Zhang J., Wang J., Wu Y. (2012). An improved approach for accurate and efficient calling of structural variations with low-coverage sequence data. BMC Bioinformatics.

[21] Rausch T., Zichner T., Schlattl A., Stütz A.M., Benes V., Korbel J.O. (2012). DELLY: structural variant discovery by integrated paired-end and split-read analysis. Bioinformatics.

[22] Zhang Z., Wang J., Luo J., Ding X., Zhong J., Wang J. (2016). Sprites: detection of deletions from sequencing data by re-aligning split reads. Bioinformatics.

[23] Safavi-Hemami H., Li Q., Jackson R.L., Song A.S., Boomsma W., Bandyopadhyay P.K. (2016). Rapid expansion of the protein disulfide isomerase gene family facilitates the folding of venom peptides. Proc Natl Acad Sci U S A.

[24] Houston N.L., Fan C., Schulze J.-M., Jung R., Boston R.S. (2005). Phylogenetic analyses identify 10 classes of the protein disulfide isomerase family in plants, including single-domain protein disulfide isomerase-related proteins. Plant Physiol.

[25] Iwasaki K., Kamauchi S., Wadahama H., Ishimoto M., Kawada T., Urade R. (2009). Molecular cloning and characterization of soybean protein disulfide isomerase family proteins with nonclassic active center motifs. FEBS J.

[26] Li C.P., Larkins B.A. (1996). Expression of protein disulfide isomerase is elevated in the endosperm of the maize *floury-2* mutant. Plant Mol Biol.

[27] Kim C.S., Gibbon B.C., Gillikin J.W., Larkins B.A., Boston R.S., Jung R. (2006). The maize *Mucronate* mutation is a deletion in the 16-kDa γ-zein gene that induces the unfolded protein response. Plant J.

[28] Bolger A.M., Lohse M., Usadel B. (2014). Trimmomatic: a flexible trimmer for Illumina sequence data. Bioinformatics.

[29] Langmead B., Salzberg S.L. (2012). Fast gapped-read alignment with Bowtie 2. Nat Methods.

[30] Li H. (2011). A statistical framework for SNP calling, mutation discovery, association mapping and population genetical parameter estimation from sequencing data. Bioinformatics.

[31] Koboldt D.C., Zhang Q., Larson D.E., Shen D., McLellan M.D., Lin L. (2012). VarScan 2: somatic mutation and copy number alteration discovery in cancer by exome sequencing. Genome Res.

[32] Krzywinski M., Schein J., Birol I., Connors J., Gascoyne R., Horsman D. (2009). Circos: an information aesthetic for comparative genomics. Genome Res.

[33] Chiang C., Layer R.M., Faust G.G., Lindberg M.R., Rose D.B., Garrison E.P. (2015). SpeedSeq: ultra-fast personal genome analysis and interpretation. Nat Methods.

[34] Davidson R.M., Hansey C.N., Gowda M., Childs K.L., Lin H., Vaillancourt B. (2011). Utility of RNA sequencing for analysis of maize reproductive transcriptomes. Plant Genome.

[35] Wallace J.C., Lopes M.A., Paiva E., Larkins B.A. (1990). New methods for extraction and quantitation of zeins reveal a high content of γ-zein in modified *opaque-2* maize. Plant Physiol.

