# Supplementary material for: An Exome-seq Based Tool for Mapping and Selection of Candidate Genes in Maize Deletion Mutants
Source: Genomics Proteomics Bioinformatics. 2019 Feb 8;16(6):439–50. doi: 10.1016/j.gpb.2018.02.003 (PMC6411947; doi:10.1016/j.gpb.2018.02.003)
Supplement: Supplementary Table S2 [file mmc2.docx]

**Table S2** **Primers used for genomic PCR verification of deletions in the maize mutants examined in the current study**

| **Mutant ID** | **Gene ID** | **Primer name** | **Primer sequence (5'-3')** |
| --- | --- | --- | --- |
| M531 | GRMZM2G162382 | E2F | GAACACCGGCGGGAAGAGGC |
| M531 | GRMZM2G162382 | E1R | GGCAAACCGCTCCCAGTCCC |
| M531 | GRMZM5G841619 | E5F | TGCTATTGACAACACCGACTACACCA |
| M531 | GRMZM5G841619 | E6R | TGCCGCTTACATTTGCACGTGT |
| M531 | AC208337.2_FG003 | E9F | GGCTCTTGAGCTTGCGCCCA |
| M531 | AC208337.2_FG003 | E8R | GCGGAGAGCATTGTAGCCAAGGG |
| M531 | GRMZM2G015534 | E5F | CACAGCTAAAAGCCGAGAATTCTTG |
| M531 | GRMZM2G015534 | E6R | CCAAACGGAACACTCATGGTTAATT |
| M531 | GRMZM5G864001 | E4F | TGTGGGCAGCCGTCTCCTCT |
| M531 | GRMZM5G864001 | E3R | AGAGGCGCAAGGTTGTCACCA |
| M523 | GRMZM2G017792 | E2F | CGTGGTCGAGGTGCCGCATC |
| M523 | GRMZM2G017792 | E1R | GCCTCCCGTCATGCCCATCG |
| M523 | GRMZM2G141273 | E1F | TCGGACGGCGTCGAGTCTGT |
| M523 | GRMZM2G141273 | E3R | TCGGGTACGCCTTCTTTGCTGC |
| M523 | GRMZM2G141386 | E6F | TCGTGCCTTCAGCTGCAGCC |
| M523 | GRMZM2G141386 | E5R | GGGTCCTACCTGCAGGGGCA |
| M523 | GRMZM2G443655 | E1F | TGACACCAACGTGAAACTGGATGC |
| M523 | GRMZM2G443655 | E1R | AGCGTTGGGTGATGCGCAAGT |
| M523 | GRMZM2G073628 | E2F | ACACCAGGGGACGCAGAATTGC |
| M523 | GRMZM2G073628 | E1R | TCGCTCACCGTCCTCGTGGT |
| M523 | GRMZM2G114354 | E1F | AGGGGCGGGTTGAGGTCCTC |
| M523 | GRMZM2G114354 | E1R | TCGCCACCCCACACACCTCC |
| M523 | GRMZM2G380955 | E1F | TGCCGCCGGGATACTCACCA |
| M523 | GRMZM2G380955 | E1R | GCGCCGATGAGCCATTTGCG |
| M1486 | GRMZM2G098603 | E5F | GCCTCTGTCTGGCAGCTGATCG |
| M1486 | GRMZM2G098603 | E3R | CCAGCACATCGAGACAGTACACCA |
| M1486 | GRMZM2G098603 | E3F | TGGTGTACTGTCTCGATGTGCTGG |
| M1486 | GRMZM2G098603 | E1R | GTCGTCCGGGTCAAGCGCAA |
| M1486 | GRMZM2G098596 | E1F | CTCACCGTCAACCCCACCGC |
| M1486 | GRMZM2G098596 | E2R | CGGTACACGTTCCACCTGCCG |
| M1486 | GRMZM2G176546 | E1F | TGGCACTACTGGCACACCGA |
| M1486 | GRMZM2G176546 | E3R | GGCAACACCACAGTGCAGTGC |
| M1486 | GRMZM2G176546 | E4F | TCTGGCTGCTTTGAGACCGACA |
| M1486 | GRMZM2G176546 | E5R | GCCCACCAGACTCCTTCACAGC |
| M759 | GRMZM2G536584 | I1F | TGCCAACAAAATCCTAGCACTGCCA |
| M759 | GRMZM2G536584 | E9R | CCCTGAAGGTCTCGACATGCACT |
| M759 | GRMZM2G089484 | E1F | GTGATCCGCCCCTTGTCGGC |
| M759 | GRMZM2G089484 | E2R | CGCCGTAGCTGCCTTTGCCT |
| M759 | GRMZM2G060987 | E3F | TGACATCTGGGCTGTGGATGGT |
| M759 | GRMZM2G060987 | E5R | ACAGGTGTTGGCCCCAGCTCT |
| M759 | GRMZM2G097289 | E6F | GACAGTGTCCGCCGTCTGCA |
| M759 | GRMZM2G097289 | E5R | TGCGTCCATTTTTGCTGAGGAGGA |
| M56 | GRMZM2G138632 | E5F | TCAGGTTCCAGCTCCAGCACT |
| M56 | GRMZM2G138632 | E4R | TCAGCCTTGCGTGGTTGGTCA |
| M56 | GRMZM2G019450 | E2F | AGGGTTCGTGATGTTGCACCAA |
| M56 | GRMZM2G019450 | E3R | AGCATGGCATGGTCTACTTGGCA |
| M56 | GRMZM2G160174 | E3F | TGGGGCGAACCAGGCTGAGG |
| M56 | GRMZM2G160174 | E2R | GCTGGAACTGACGGAGATCAGGG |
| M56 | GRMZM2G407044 | E8F | TCCAGAACACCCCAATGAGCATCA |
| M56 | GRMZM2G407044 | E7R | CCCAGACCTGATAGATGCGTTGCC |
| M56 | GRMZM2G030125 | I1F | GCGAGGAGCAGGCCGATCAC |
| M56 | GRMZM2G030125 | E1R | AATGCCACGCCGGATGCCAA |
| M56 | GRMZM2G044677 | E1F | TGTGGGCGCCGTGTCTTCAC |
| M56 | GRMZM2G044677 | E1R | CTGTCGCGAGCACCAGAGGC |
| M1338 | GRMZM5G852911 | E6F | CGAGCTGTTCTCTCGCCTCGT |
| M1338 | GRMZM5G852911 | I6R | ACCTTGAAGCTGGAGACGAGGCA |
| M1338 | GRMZM2G045668 | E7F | TGCTTCTTGTCATCTCCGGCATCA |
| M1338 | GRMZM2G045668 | I6R | TGCAACATTTTGGAGGTGATTGCATGT |
| M1338 | GRMZM2G118743 | E3F | GCTTCCCAAACCCGTGGCGA |
| M1338 | GRMZM2G118743 | I4R | AGGAGCCAAGATCCTCCTTGCA |
| M1338 | GRMZM2G420723 | E2F | GGACAAGCCCAAATTCATTGCATCC |
| M1338 | GRMZM2G420723 | E1R | TGAGTGGGGTGTCAGGCCGA |
| M1338 | GRMZM2G126656 | E13F | TGTCAGCAGCACTCGGTCACC |
| M1338 | GRMZM2G126656 | E10R | TGCAGAGGTTCTGGGACTCTGCT |
| M183 | GRMZM2G435475 | E1F | ACATGCTGCCGCCTCACGTC |
| M183 | GRMZM2G435475 | E1R | GCTCCCCAAAAGGACTTACACAACC |
| M183 | GRMZM2G044368 | E2F | TGGGACACTGCTGGCCAGGA |
| M183 | GRMZM2G044368 | E4R | TCCCTTTGGAAGCACACCATGC |
| M183 | GRMZM2G168898 | E4F | TGTCCAGCAGCGCCGTCTTG |
| M183 | GRMZM2G168898 | E2R | GCCGGGTGCCAAGCAGATGT |

*Note*: Primers are named according to their locations in the respective genes, with embedded number representing the involved exon or intron number; E for exon and I for intron, F for forward primer and R for reverse primer. Primers were designed based on the maize reference genome (RefGen_v3.25).
